# Midline Catheters: A Novel Curriculum for Emergency Medicine Residents

**DOI:** 10.5070/M5.52243

**Published:** 2026-04-30

**Authors:** Braden W McIntosh, Brian Allen, Michael Truax, Mitchell Hymowitz, Greggory Davis

**Affiliations:** * Louisiana State University Health Sciences Center, Department of Emergency Medicine, Baton Rouge, LA

## Abstract

**Audience and Type of Curriculum:**

This curriculum is designed for all levels of emergency medicine (EM) Residents. The curriculum covers the appropriate anatomy, indications, contraindications, and specific steps for placement of midline catheters.

**Length of Curriculum:**

Three one-hour sessions over a period of three months or a single three-hour session.

**Introduction:**

Midline catheters have become more mainstream in the emergency department as a replacement for central lines. They are readily insertable into a peripheral vein and useful for access, blood draws, and medication administration, which are well known benefits of peripheral lines. They also offer long-term reliable access and are becoming more accepted for caustic medication administration, which are clear indications for central line placement. They are significantly more comfortable than a central line and may be associated with lower rates of catheter related bloodstream infections (CRBSI) when compared to peripherally inserted central catheter (PICC) lines.^1^ These combined advantages have led to increasing interest in midline use in the emergency department. Unfortunately, EM residents are often not educated on the indications for midline catheters and are not trained in placing them either. We propose a midline curriculum for EM resident success in this area.

**Educational Goals:**

The purpose of this curriculum is to teach emergency medicine residents how to place and utilize midline catheters.

**Educational Methods:**

The educational strategies used in this curriculum include written and skills assessments as well as in-person lectures, and asynchronous learning. Specifically, there is a pre- intervention and post-intervention knowledge assessment as well as a pre- and post-skills assessment and skills check list. The knowledge assessments are conducted using written multiple-choice exams, and the skills curriculum evaluation is performed with task trainers and instructor feedback.

**Research Methods:**

Educational content was evaluated by learners via online survey. Efficacy of the educational content was assessed using scores and feedback from the written pre- and post-intervention examinations and procedural skills assessments.

**Results:**

Thirty-nine residents completed the study curriculum. Overall, we found the written exam score improved from pre- to post-intervention by roughly 5% (75% to 80% on average). The time to midline placement from tourniquet to vessel catheterization was significantly improved from pre-intervention at 7 minutes, 32 seconds to post-intervention at 5 minutes, 0 seconds. The post-curriculum survey taken by participants demonstrated an increased self-reported likelihood of placing a midline on a patient and improved self-reported core knowledge. However, self-reported clinical skills did not improve significantly.

**Discussion:**

The intent of our midline curriculum was to improve EM resident knowledge regarding line usage, placement, and confidence in using them in practice. The EM residents’ knowledge, ability to place, and self-reported intent to use midlines more often was improved based on our pre/post written, skills assessments and our feedback sessions. This project was well received and will hopefully result in more midline placement in the emergency department which should benefit learners, nurses, and patients.

**Topics:**

Midline placement, curriculum, emergency medicine, procedural skills assessment.

## USER GUIDE

**Table:** 

List of Resources:	
Abstract	1
User Guide	3
Didactics and Hands on Curriculum Chart	7
Appendix A: Resident Midline Pre-/Post-Intervention Examinations	9
Appendix B: Clinical Skills Assessment Tool: Ultrasound Guided Midline Catheterization-Post Intervention	18
Appendix C: Intervention PowerPoint	24
Appendix D: Midline Education Evaluation	25

**Learner Audience:**

This curriculum is appropriate for all PGYs.

**Length of Curriculum:**

This curriculum can be completed in three one-hour long sessions. These could be completed all in one day, but more realistically, split into one session per month over three months is ideal. There is no pre-reading required to determine raw knowledge prior to assessment. The pre-and post-intervention knowledge assessment as well as the video lectures can be completed asynchronously or synchronously. The skills assessments must be completed synchronously in person. The pre-and-post knowledge assessment time allotment is approximately 30 minutes each, and the final skills lab time allotment is approximately 30 minutes. Thus, the final hour of this curriculum allots 30 minutes for the knowledge assessment and 30 minutes for the skills assessment.

**Topics:**

Midline placement, curriculum, emergency medicine, procedural skills assessment.

**Objectives:**

Upon completion of this curriculum, the learner should be able to:

1. Identify the uses and advantages of midline catheters.
2. Be facile in the placement of midline catheters.

### Brief introduction

Midline catheters are peripherally inserted venous access lines, usually placed in a patient’s upper extremity a few inches above the antecubital fossa using ultrasound guidance in either the basilic, cephalic, or brachial veins.^2^ Fixed length catheters are tunneled into the skin 8–12 centimeters (cm) and trimmable midline catheters require measuring from the desired insertion point to the axilla and cutting it to length so that the tip of the catheter in the axillary vein is located slightly distal to the axilla when inserted. Midline catheters have gained popularity in previous years because they offer an intermediate between peripheral and central lines while combining some of the benefits conferred by both types of intravenous access.^3^,^4^ They are readily insertable into a peripheral vein and useful for access, blood draws, and medication administration, which are well known benefits of peripheral lines. They also offer longer-term reliable access and are becoming more accepted for administration of pressors and short-term antibiotics,^5^ which are clear indications for central line placement. Additionally, they may be associated with lower rates of catheter related bloodstream infections (CRBSI) when compared to peripherally inserted central catheter (PICC) lines.^1^,^6^ While there is currently no conclusive morbidity or mortality data supporting the use of midlines, their use may become much more common as hospitals make concerted efforts to reduce CRBSI’s and their associated morbidity.^7^,^8^

Given these advantages, it is reasonable to assume that midline catheter placement in the emergency department may find a place in the standard of care for patients who have difficult access or will likely require intensive care unit (ICU) admission. As such, many residency programs have made concerted efforts to incorporate midline placement training into their curriculum, with varying degrees of success. There is currently no standardized curriculum for teaching midline placement, and as a result, resident comfort with midline placement varies widely both within and between programs. This disparity is important, especially given that many complications of IV lines, including arterial placement, mispositioning, and bacterial introduction are ultimately the result of provider error and can carry significant morbidity.^9^ Programs have developed standardized curricula for teaching central line placement best practices with much success,^10^ but currently no such curriculum exists for midline placement. This lapse in training may lead to worse patient outcomes, necessitating the development of a standardized curriculum for midline placement as part of emergency medicine residency programs. This paper provides a framework for an evidence-based curriculum instructing midline insertion.

### Problem identification, general and targeted needs assessment

The purpose of this study is to produce evidence-based interventions that could later be incorporated into a standardized curriculum for midline placement didactics and skills labs. The ultimate goal of this curriculum is to train future physicians on how to safely and proficiently place midlines. We therefore identified primary outcome measures as number of errors and time to completion on a training model. Additionally, these models are standardized, universally available, and negate the potential risk to patients that is posed from training only in the department. This also provides the advantage of offering streamlined training, wherein instructors can incorporate didactics and clinical assessments into a single training session with real-time feedback. Since most programs have dedicated time for didactics and simulation labs, this curriculum is optimized for training residents on midlines efficiently.

Many of the indications, techniques, and precautions for midline placement are shared with those of central line placement. Given these similarities, we aimed to build our curriculum model on the evidence-based training methods surrounding central lines that many programs have already incorporated with success. Meta-analyses have reliably demonstrated that technology-enhanced simulation with real-time feedback improve residents clinical and practical decision-making skills when successfully incorporated into curricula.^11^,^12^ For instance, it has already been demonstrated that simulation training for central line placement both improves success rate and reduces complication rates.^13^,^14^ Additionally, incorporation of asynchronous learning into didactics has been well-received by residents and is non-inferior to in-person didactic sessions.^15^ Asynchronous learning also proved an invaluable tool during the COVID-19 pandemic because it lends very well to education via e-modules and virtual conferences. Specifically, knowledge assessments and lectures with asynchronous options provide a flexible learning environment for residents.

Given that many of the interventions of this study’s proposed midline curriculum (eg, simulation training, real-time feedback, asynchronous learning) have already shown universal utility and have been effectively implemented for central lines, it is reasonable to apply these interventions and anticipate them to be equally useful when developing a midline curriculum. However, to the authors’ knowledge, there is currently no literature that investigates the specifics of how these training techniques can most effectively be implemented for midline placement.

### Goals of the curriculum

Establishing rapid intravenous access to facilitate resuscitation and administer critical medications is fundamental to the practice of emergency physicians.^16^ Midline placement has come to the forefront as an access modality that could provide a combination of benefits associated with both peripheral and central access catheters.^17^ As the use of midlines expands in the practice of emergency medicine, familiarity with these devices is paramount to ensure proper implementation and utility. A fitting place for focusing on education begins with emergency physicians in training, namely those currently in emergency medicine residency programs. The primary goals of this curriculum are to strengthen resident learner knowledge of the indications for midline catheters and improve technical skills in device placement.

### Objectives of the curriculum

Upon completion of this curriculum, the learner should be able to:

1. Identify the uses and advantages of midline catheters.
2. Be facile in the placement of midline catheters.

### Educational Strategies

For session one, learners were first given a 24-question multiple-choice pre-test to evaluate base-line knowledge of midline catheters. This exam was in the format of an online Google form. Learners were given as much time to complete it as necessary. After completion of the written exam, learners were then brought to the simulation laboratory for procedural skill assessment. Learners were told they needed to place a midline catheter using full-sterile technique similar to a central line. In front of them were all the necessary components of the midline catheter kit as well as all the components for full sterile placement of the line. Learners were encouraged to familiarize themselves with all the components of the kit. A ready to use ultrasound was made available as well. Learners were then asked to place a tourniquet and identify the target vessel on the live instructor and then directed to place the midline on the task trainer. One of the measured time points for the study is start to completion; start time was recorded once the tourniquet was placed by the learner onto the instructor. Once the vessel was determined, the learner then began the midline placement procedure on a task trainer developed for midline placement using Humimix gel.

The instructors had a 27-point check list with which to grade the learner and kept track of the amount of time required for successful placement of the catheter. Once the learner had completed the placement of the catheter, he or she was given immediate feedback as to the steps he or she had missed. Learners were then given time to manipulate the contents of the kit as desired, and any confusing components of the procedure were modeled by the instructor as requested by the learner.

Following the procedural skills assessment, learners were given a thirty-minute PowerPoint presentation. This could be done in person but was also recorded to be viewed asynchronously as needed. Following the lecture, the knowledge and procedural skills assessments were repeated to assess for improvement. This was followed by a final evaluation to determine the effectiveness of the curriculum.

### Results and tips for successful implementation

Thirty-nine residents completed the study curriculum.

Residents were pulled from lecture during a normal conference day to complete their sessions. Thirty-three-point three percent (n = 13) were post graduate year (PGY)1s, 43.6% (n = 17) were PGY2s, and 23.1% (n = 9) were PGY3s. We collected data over a period of 18 months. Overall, we found the score on the written exam to improve from an average of 75% to 80% with p value of 0.012. (See supplemental information for the written exam.) Regarding clinical skills placement, we found the time to midline placement from tourniquet to vessel catheterization was significantly improved from 7 minutes 32 seconds pre-intervention to 5 minutes 0 seconds post-intervention with p value <0.001. Arterial puncture occurred once pre-intervention and twice post- intervention. This was not statistically significant, p > 0.9. (See supplemental information for clinical skills checklist.)

The post-curriculum survey had a completion rate of 46%. Thirty-three-point three percent were PGY1, 27.8% were PGY2, 38.9% were PGY3. Average core knowledge prior to the curriculum was rated as a 1.61 out of 5 with standard deviation of 0.61. Average procedural competence prior to the curriculum was rated as a 1.94 out of 5 with standard deviation of 0.87. Likelihood to place a midline catheter prior to the curriculum was a 1.17 out of 5 with a standard deviation of 0.51.

Effectiveness of the curriculum in regard to teaching core knowledge was rated as a 4.17 out of 5 with standard deviation of 0.62. Effectiveness of the curriculum regarding clinical skills was rated as a 4.11 out of 5 with standard deviation of 0.58. Finally, likelihood to place a midline catheter after completing the curriculum was a 3.72 out of 5 with standard deviation of 0.83.

The pre-curriculum survey showed that when asked about their likelihood of placing a midline on a patient, 89% ( n = 16) of residents rated themselves as a 1 on a scale of 1–5, with 1 being “unlikely to place a midline,” and 5 being “already placing midlines on patients,” with one resident rating themselves as a 2, and one resident rating themselves as a 3.

After training, 17% of residents (n = 3) rated themselves as a 5, 44% of residents (n = 8) rated themselves as a 4, 33% (n= 6) as a 3, and 5.6% (n= 1) as a 2, p < 0.01. Self-identified core knowledge, on a scale of 1–5, with 1 being “knew nothing” and 5 being “expert knowledge,” residents went from 50% (n = 9) rating themselves as a 2 and 44% (n = 8) rating themselves as a 1 prior to the curriculum, to 28% (n = 5) rating themselves as a 5, 61% (n = 11) rating themselves as a 4, and 11% (n = 2) rating themselves as a 3 post-curriculum, p=0.01.

Finally, using the same five-point scale, self-identified clinical skills went from 33% (n = 6) rating themselves as a 1, 44% (n = 8) rating themselves as a 2, and 17% (n = 3) rating themselves as a 3 prior to the curriculum, to 22% (n = 4) rating themselves as a 5, 67% (n = 12) rating themselves as a 4, and 11% (n = 2) rating themselves as a 3 post-curriculum, p = 0.54.

It is worth noting that this curriculum was designed based upon the use of POWERWAND midline catheters. Educators should modify slides 19, 20, 36, and 38 to reflect the use of different catheter brands as appropriate.

### Evaluation and Feedback

A video of the lecture was created for learners to view at their own pace if they were unable to complete the in-person lecture. It was also suggested to add a sharps container to improve realism and to add a soap dispenser at workstation to increase the likelihood of hand washing.

### Associated Content

Appendix A: Resident Midline Pre-/Post-Intervention Examinations

Appendix B: Clinical Skills Assessment Tool: Ultrasound Guided Midline Catheterization-Post Intervention

Appendix C: Intervention PowerPoint/Video (http://bit.ly/4cPFz3g or https://youtu.be/mJG_-UYLmM0)

Appendix D: Midline Education Evaluation

## Supplementary Information





## Figures and Tables

**Table:** 

Topic	Recommended Educational Strategy	Educational Content	Objectives	Learners	Timing, Resources Needed	Recommended Assessment, Milestones Addressed
**Pre-instruction Midline Knowledge Assessment**	Pre-instruction 24 Question Multiple Choice assessment *Can be completed asynchronously.	Residents are exposed to questions regarding ultrasound, ultrasound guided IVs, midline pertinent anatomy, indications and contraindications for midlines, and placement technique.	To assess residents baseline knowledge of midline catheters and placement.	All PGYs	30-minute Pre-assessment: Instructor(s): 1 Equipment: Paper / Online Survey Format *Can be done asynchronously.	Milestone: MK1, MK2 Assessment: Pre instruction multiple choice test. See Appendix-A.
**Pre-instruction Midline Skills Assessment**	Pre-instruction Procedural Skills Assessment Hands on-Instructor proctored. - Residents place midline with no baseline knowledge using a 31-point check list. Keep track of time to successful placement of catheter. Give immediate feedback to residents upon completion of catheter placement.	Familiarize themselves with all components of the midline kit. Place a tourniquet and identify the target vessel on the live instructor. Place the midline on the task trainer. Place a midline catheter using full-sterile technique similar to a central line.	To expose residents to all components of midline kit and attempt placement with no prior knowledge.	All PGYs	30-minute Pre-instruction Procedural Skills Assessment: Instructor(s): 1 per resident Equipment: 1 US machine per resident; 1 task trainer per resident; 1 midline kit per resident (can be re-used).	Milestone: PC8 Assessment: - Checklist Real-time feedback by an instructor. See Appendix-B.
**Midline Catheter Lecture**	PowerPoint Lecture-approximately 30 minutes. Lecture to be given by the educator or can be watched asynchronously with included video presentation.	Advantages and disadvantages of midline catheters Indications/ Contraindications for use. Components of kit. Pertinent Anatomy. Placement Strategy.	To Identify the uses and advantages of midline catheters. To demonstrate placement of midline catheters.	All PGYs	30-minute PowerPoint Lecture: Instructor(s): 1 Equipment: Computer *Video recording optional which can be viewed asynchronously.	Milestone: MK1, MK2, PC8 See appendix C.
**Post-instruction Midline knowledge Assessment**	24 Question Multiple Choice assessment *Can be completed asynchronously.	Residents are again exposed to questions regarding ultrasound, ultrasound guided IVs, midline pertinent anatomy, indications and contraindications for midlines, and placement technique.	To evaluate residents’ retention of knowledge of midline catheters and placement.	All PGYs	30-minute Post-assessment: Instructor(s): 1 Equipment: Paper / Online Survey Format *Can be done asynchronously.	Milestone: MK1, MK2 Assessment: Post-instruction Multiple Choice Test. See Appendix-A.
**Post-instruction Midline skills Assessment**	Post-instruction Procedural Skills Assessment Hands on-Instructor proctored. - Residents place midline with no baseline knowledge using a 31-point check list. Keep track of time to successful placement of catheter. Give immediate feedback to residents upon completion of catheter placement.	Refamiliarize themselves with all components of the midline kit. Place a tourniquet and identify the target vessel on the live instructor again. Place the midline on the task trainer again. Place a midline catheter using full-sterile technique similar to a central line again.	To demonstrate expertise in placing a midline catheter after having gained prior knowledge and hands-on exposure to midline catheters.	All PGYs	30-minute Post-intervention Procedural Skills Assessment: Instructor(s): 1 per resident Equipment: 1 US machine per resident; 1 task trainer per resident; 1 midline kit per resident (can be re-used).	Milestone: PC8 Hands-On: Checklist for grading. See Appendix-B.
**Post Midline Curriculum Survey**	Post-Curriculum Survey *Can be taken asynchronously.	n/a	To determine the effectiveness of midline curriculum. To determine anticipated future utilization of midline catheters.	All PGYs	5-minute Survey: Instructor(s): 1 Equipment: Paper / Online Survey Format *Can be done asynchronously.	Milestone: MK1,MK2 PC8 Assessment: Survey see Appendix-D.

**Figure:**
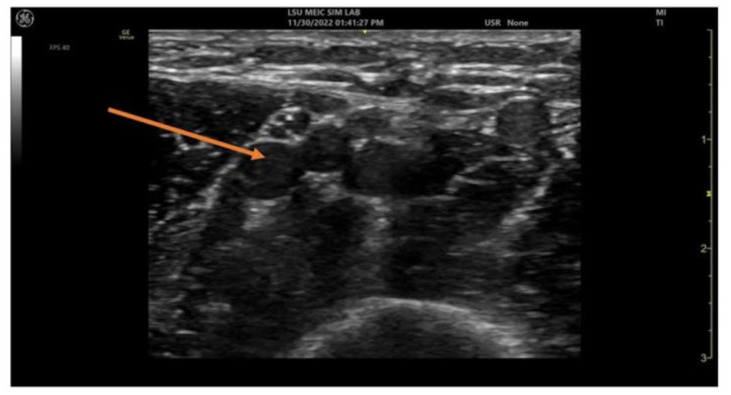
Author’s own image

**Figure:**
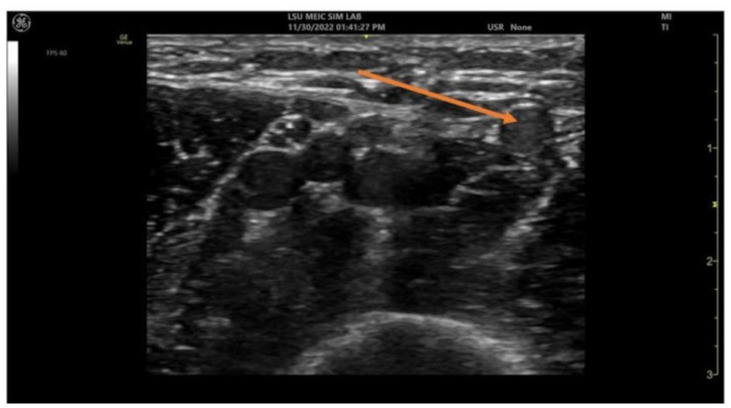
Author’s own image

**Figure:**
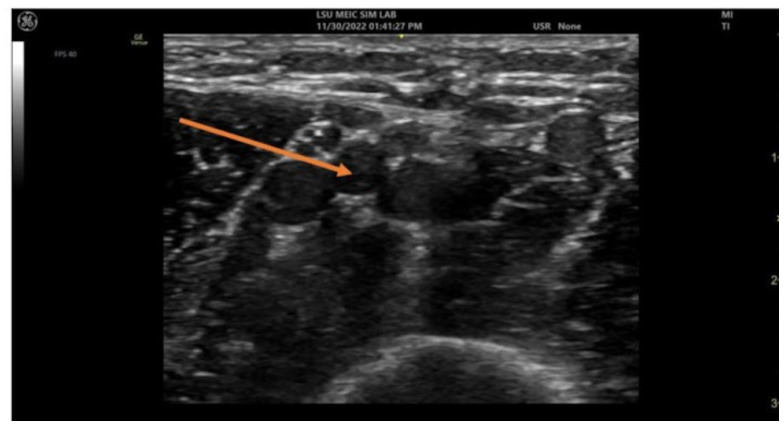
Author’s own image

**Figure:**
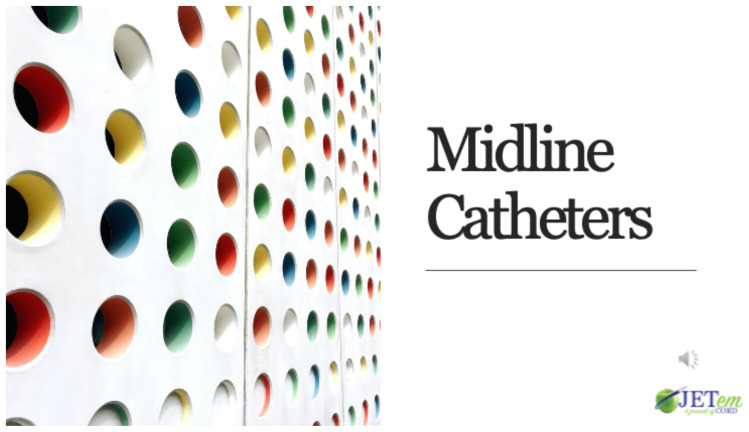
Please see associated PowerPoint and video files (large files so alternate download options below): PowerPoint Link: http://bit.ly/4cPFz3g Video Link: https://youtu.be/mJG_-UYLmM0

## Appendix A Resident Midline Pre-/Post-Intervention Examination

^*^Indicates required question

1. Enter your name here^*^________________________________________
2. Please select your PGY level at the time of start of the midline curriculum
  - □ PGY 1
  - □ PGY 2
  - □ PGY 3
  - □ PGY 4
3. Which of the following is characteristic of the sonographic appearance of a vein?
  Mark only one.
  - □ Thin walls
  - □ Pulsatile
  - □ Irregular walls
  - □ Thick walls
4. Which of the following is the most appropriate choice of transducer for vascular access?
  Mark only one.
  - □ Curvilinear
  - □ Linear transducer
  - □ Microconvex transducer
  - □ High frequency linear / hockey-stick
5. Before the ultrasound transducer may be placed on the sterile field it must:^*^
  Mark only one.
  - □ Be autoclaved
  - □ Be covered with a sterile transducer cover
  - □ Be prepped with betadine
  - □ Be washed with warm soapy water
6. During a scan of the arm, the vein may be differentiated from the artery by:^*^
  Mark only one.
  - □ Compressibility of the venous walls
  - □ Identification of pulsatile flow in the vein
  - □ Inability to compress the venous walls
  - □ Observation of phasic flow in the artery
7. In order to reduce the risk of infection, which steps should be performed before/during midline placement?^*^
  Mark only one.
  - □ Ensure probe is thoroughly cleaned with US wipes
  - □ Prep with chlorhexidine
  - □ Use full barrier precautions
  - □ Wash your hands
  - □ All of the above
8. Which of the following is a drawback of the transverse technique of line placement?
  Mark only one.
  - □ Visualization of the brachial artery
  - □ Visualization of the brachial vein
  - □ Visualization of the needle tip
  - □ Visualization of the biceps radialis
9. What is the structure represented by the arrow? ^*^
  Mark only one.
  - □ Brachial Artery
  - □ Brachial vein
  - □ Cephalic vein
  - □ Basilic vein
  - □ Median Nerve
10. Which technique of midline placement allows for visualization of the anatomy surrounding the brachial vein and visualization of the entire needle? ^*^
  Mark only one.
  - □ Landmark technique
  - □ Longitudinal technique
  - □ Oblique technique
  - □ Transverse technique
11. Which of the following would be appropriate sites to place a midline catheter? ^*^
  Mark only one.
  - □ Basilic vein
  - □ Brachial vein
  - □ Cephalic vein
  - □ Axillary vein
  - □ All of the above
12. Which of the following steps should be performed first during midline placement? ^*^
  Mark only one.
  - □ Conduct a procedural timeout
  - □ Obtain written informed consent
  - □ Place a sterile cover on the transducer
  - □ Prep the area with chlorhexidine
13. For midlines that can be cut to approximate length, where should the tip of the catheter ideally terminate once appropriately placed?
  Mark only one.
  - □ Antecubital fossa
  - □ Inferior neck
  - □ Just distal to the axilla
  - □ Forearm
14. What is the potential benefit of using a midline that can be cut to determine catheter length?
  Mark only one.
  - □ Ensure that the midline terminates in a central vein
  - □ Facilitate easier catheter insertion
  - □ Improve device securement after catheter placement
  - □ Ability to measure and accommodate for individual patient anatomy
15. What is the structure represented by the arrow? ^*^
  Mark only one.
  - □ Brachial Artery
  - □ Brachial vein
  - □ Cephalic vein
  - □ Basilic vein
  - □ Median Nerve
16. When using color Doppler to evaluate flow, what does red flow represent? ^*^
  Mark only one.
  - □ Flow away from the transducer
  - □ Flow toward the transducer
  - □ High velocity flow
  - □ Low velocity flow
17. After obtaining informed consent and positioning the patient, what is the next step in ultrasound guided midline placement? ^*^
  Mark only one.
  - □ Establishing a sterile field
  - □ Pre-procedure chest radiograph
  - □ Pre-procedure antibiotic administration
  - □ Ultrasound survey of the potential site
18. When using color Doppler to evaluate flow, what does blue flow represent? ^*^
  Mark only one.
  - □ Flow away from the transducer
  - □ Flow toward the transducer
  - □ Venous flow
  - □ Arterial flow
19. What is the structure represented by the arrow? ^*^
  Mark only one.
  - □ Brachial Artery
  - □ Brachial vein
  - □ Cephalic vein
  - □ Basilic vein
  - □ Median nerve
20. Which of the following are potential complications of cannulation of the brachial artery?
  Mark only one.
  - □ Blood clot
  - □ Tissue necrosis
  - □ Infection
  - □ Air embolus
  - □ All of the above
21. After the line is properly secured and flushed, what is the next step in midline catheter placement?^*^
  Mark only one.
  - □ No further steps are necessary
  - □ Obtain a chest radiograph to confirm placement
  - □ Perform a blood gas to verify placement
  - □ Thoroughly scrub the area with soapy water
22. Advantages of ultrasound-guided midline placement over the traditional ultrasound-guided peripheral line include which of the following?
  Mark only one.
  - □ Decreased infection rate
  - □ Decreased line failure
  - □ Increased life of the line
  - □ Ability to administer pressors
  - □ All of the above
23. After suturing the midline in place, which type of dressing should be applied?
  Mark only one.
  - □ No dressing is necessary
  - □ Silk tape
  - □ Transparent dressing
  - □ Sterile gauze
24. Which of the following is most helpful in determining inadvertent arterial placement of a catheter?
  Mark only one.
  - □ Color of blood return
  - □ Pulsatility of blood return
  - □ Transduction of vascular pressure
  - □ Radiograph appearance

## Appendix B Clinical Skills Assessment Tool: Ultrasound Guided Midline Catheterization-Post Intervention

Resident Name: _________________________________

Date: _____________

PGY Year: __________

Evaluator: _____________________________________

1. Obtain informed consent?
  Mark only one.
  □ yes
  □ no
2. Start time (tourniquet time on evaluator): _____________
3. Perform pre-procedure scan of site?
  Mark only one.
  □ yes
  □ no
4. Wash Hands
  Mark only one.
  □ yes
  □ no
5. Trimmable catheters only - Measures from insertion site to just distal to the axilla
  Mark only one.
  □ yes
  □ no
6. Trimmable catheters only - Cut catheter
  Mark only one.
  □ yes
  □ no
7. Prep with chlorhexidine
  Mark only one.
  □ yes
  □ no
8. Operator uses full barrier precautions
  Mark only one.
  □ yes
  □ no
9. Drape patient using sterile technique
  Mark only one.
  □ yes
  □ no
10. Sterile transducer cover applied to ultrasound
  Mark only one.
  □ yes
  □ no
11. Pre-procedure “timeout”
  Mark only one.
  □ yes
  □ no
12. Ultrasound identification of brachial or basilic vein
  Mark only one.
  □ yes
  □ no
13. Anesthetize site
  Mark only one.
  □ yes
  □ no
14. Dynamic ultrasound insertion of needle
  Mark only one.
  □ yes
  □ no
15. Seldinger technique
  Mark only one.
  □ yes
  □ no
16. Trimmable catheter only - Skin nick (per manufacturer instructions)
  Mark only one.
  □ yes
  □ no
17. Trimmable catheter only - Insert dilator (per manufacturer instructions).
  Mark only one.
  □ yes
  □ no
18. Trimmable catheter only - Remove dilator (per manufacturer instructions). If breakaway dilator, insert catheter through dilator prior to breakaway.
  Mark only one.
  □ yes
  □ no
19. Secure catheter
  Mark only one.
  □ yes
  □ no
20. Finish time (once catheter is in skin): _____________
21. Transparent dressing
  Mark only one.
  □ yes
  □ no
22. Discard sharps
  Mark only one.
  □ yes
  □ no
23. Number of Attempts:
  Mark only one.
  □ 1
  □ 2
  □ 3
  □ 4
  □ 5
  □ 6
24. Skin to vein time (start minus stop time, can calculate later): _____________
25. Brachial artery puncture?
  Mark only one.
  □ yes
  □ no
26. Ultrasound Approach:
  Mark only one.
  □ transverse
  □ longitudinal
  □ oblique
  □ combined

## Appendix D Midline Education Evaluation

To evaluate the quality of the educational materials provided for midline education.

^*^Indicates required question

Enter your name here^*^________________________________________

1. Please select your PGY level at the time of completion of the midline curriculum.^*^
  - □ PGY 1
  - □ PGY 2
  - □ PGY 3
  - □ PGY 4
2. On a scale of 1–5, rate your knowledge regarding midline catheters prior to this curriculum.^*^
  Mark only one.
  **Table:**
  1	2	3	4	5
  □	□	□	□	□
  Knew nothing				Expert knowledge
3. On a scale of 1–5, please rate your procedural competence in placing midline catheters prior to completion of the midline curriculum. ^*^
  Mark only one.
  **Table:**
  1	2	3	4	5
  □	□	□	□	□
  Incompetent				Expert level
4. On a scale of 1–5, how likely were you to place a midline on a patient prior to the midline training? ^*^
  Mark only one.
  **Table:**
  1	2	3	4	5
  □	□	□	□	□
  Unlikely				Already placing midlines on patients
5. Please evaluate the effectiveness to teach you core midline knowledge of this curriculum. ^*^
  Mark only one.
  **Table:**
  1	2	3	4	5
  □	□	□	□	□
  Ineffective				Greatly effective
6. Please evaluate this curriculum’s effectiveness in teaching you the procedural skills necessary to place a midline catheter. ^*^
  Mark only one.
  **Table:**
  1	2	3	4	5
  □	□	□	□	□
  Ineffective				Greatly effective
7. On a scale of 1–5, how likely are you to place a midline on a patient now that you have completed the midline training? ^*^
  Mark only one.
  **Table:**
  1	2	3	4	5
  □	□	□	□	□
  Unlikely				Very likely
8. Will place a midline at next opportunity if not already placing midlines.
  □ yes
  □ no

## References

[1] Urtecho M, Torres Roldan VD, Nayfeh T (2023). Comparing complication rates of midline catheter vs peripherally inserted central catheter. A systematic review and meta-analysis. Open Forum Infect Dis.

[2] Elli S, Pittiruti M, Pigozzo V (2020). Ultrasound-guided tip location of midline catheters. J Vasc Access.

[3] Paje D, Walzl E, Heath M (2025). Midline vs peripherally inserted central catheter for outpatient parenteral antimicrobial therapy. JAMA Intern Med.

[4] Adams DZ, Little A, Vinsant C, Khandelwal S (2016). The midline catheter: A clinical review. J Emerg Med.

[5] Hao Q, Horton J (2023). Midline and extended dwell catheters for IV antibiotics. CADTH Technol Overv.

[6] Adams DZ, Little A, Vinsant C, Khandelwal S (2016). The midline catheter: A clinical review. J Emerg Med.

[7] Selby LM, Rupp ME, Cawcutt KA (2021). Prevention of central-line associated bloodstream infections: 2021 Update. Infect Dis Clin North Am.

[8] Prasanna N, Yamane D, Haridasa N, Davison D, Sparks A, Hawkins K (2021). Safety and efficacy of vasopressor administration through midline catheters. J Crit Care.

[9] Roldan CJ, Paniagua L (2015). Central venous catheter intravascular malpositioning: Causes, prevention, diagnosis, and correction. West J Emerg Med.

[10] Teja B, Bosch NA, Diep C (2024). Complication rates of central venous catheters: A systematic review and meta-analysis [published correction appears in *JAMA Intern Med*. 2024 Jun 1;184(6):707. doi:10.1001/jamainternmed.2024.2175]. JAMA Intern Med.

[11] Alsaad AA, Bhide VY, Moss JL, Silvers SM, Johnson MM, Maniaci MJ (2017). Central line proficiency test outcomes after simulation training versus traditional training to competence. Ann Am Thorac Soc.

[12] Madenci AL, Solis CV, de Moya MA (2014). Central venous access by trainees: A systematic review and meta-analysis of the use of simulation to improve success rate on patients. Simul Healthc.

[13] Hildreth AF, Maggio LA, Iteen A, Wojahn AL, Cook DA, Battista A (2023). Technology-enhanced simulation in emergency medicine: Updated systematic review and meta-analysis 1991–2021. AEM Educ Train.

[14] Ilgen JS, Sherbino J, Cook DA (2013). Technology-enhanced simulation in emergency medicine: a systematic review and meta-analysis. Acad Emerg Med.

[15] Jameyfield EL, Tesfai S, Palma AA, Olson AS (2023). An asynchronous curriculum: Learner perspectives on incorporating asynchronous learning into in-person and virtual emergency residency didactics. Cureus.

[16] Ernstmeyer K, Christman E (2023). Administer IV Push Medications, Chapter 2. Open Resources for Nursing (Open RN). Nursing Advanced Skills [Internet].

[17] Chopra V, Flanders SA, Saint S (2015). The Michigan Appropriateness Guide for Intravenous Catheters (MAGIC): Results From a multispecialty panel using the RAND/UCLA Appropriateness Method. Ann Intern Med.

